# Current usage of explainer animations in trials: a survey of the UKCRC registered clinical trial units in the UK

**DOI:** 10.1186/s13063-024-08060-6

**Published:** 2024-03-28

**Authors:** Vicki S. Barber, Clare Calvert, Duncan Appelbe, Kirsty Sprange, Claire Nollett, Samantha Tanner, Duncan B. Richards

**Affiliations:** 1 Oxford Clinical Trials Research Unit (OCTRU), University of Oxford, Oxford, UK; 2 Nottingham Clinical Trials Unit, University of Nottingham, Nottingham, UK; 3 Centre for Trials Research, Cardiff University, Cardiff, UK

**Keywords:** Clinical trials unit, Survey, Digital tool, Explainer animation

## Abstract

**Background:**

Explainer animations are a means to communicate aspects of clinical trials to participants in a more engaging and accessible way. Delivered well these have the potential to enhance recruitment and retention. The range of media technology used to deliver this material is expanding rapidly but is highly fragmented. Usage of explainer animations across the UK is unknown, the aim of this research was to determine current usage across the 52 registered UK Clinical Research Collaboration (UKCRC) Clinical Trials Units (CTUs) to understand the current landscape and any barriers that could be preventing wider uptake of this functionality.

**Methods:**

A survey link was emailed to all UKCRC CTU Directors and Trial Management Leads to ascertain current usage of explainer animations within their CTU. The survey ran between 01 February 2023 and 07 March 2023.

**Results:**

Responses were received from 35 CTUs—representing a response rate of 67%. 24 CTUs (69%) reported that they had created/used at least one explainer animation within their unit, although the usage, cost, length and production activities varied among the units.

**Conclusions:**

The survey showed that a high proportion of the UKCRC CTUs have used explainer animations to provide information to participants about clinical studies. For those not using the technology yet, the most common reasons cited were a lack of expertise, lack of resources and costs to produce them. One of the desired outcomes of this project is the creation of a free-to-use library of animations to encourage wider uptake and avoid duplication.

**Supplementary Information:**

The online version contains supplementary material available at 10.1186/s13063-024-08060-6.

## Background

It is a core principle of clinical trials that potential participants should have a good understanding of the trial and what it will mean for them. Modern technologies have widened the range of methods available to promote this understanding. Increasing patient knowledge about standard medical procedures using animations is not new [1–4] but only recently has this technology been applied to trials. Recent reviews have reported that short animations (animated videos, also called explainer animations and referred to throughout this paper as EAs) can be an effective means to deliver information in a number of clinical and health contexts [5]. A review in 2018 of the UK Clinical Research Collaboration (UKCRC) network of Clinical Trial Units examined the use of digital tools for recruitment and retention in randomised controlled trials [6]. This found that a wide range of digital tools (22 in total) were being used, broken down into the broad categories of Database tools, Social media, Trial websites, ISRCTN, and other in-house tools—although explainer animations (EAs) were not specifically mentioned in the review. Thus, the landscape is highly fragmented inhibiting consistent uptake and potentially leading to duplication of resources.

A Cochrane review on audio-visual presentation of information for informed consent for participation in clinical trials concluded that although the evidence, which included studies that had used animations in both videotape, computer disk and web-based animations remains largely unclear, trends were starting to emerge with regard to improvements in participant knowledge and satisfaction when the review was published in 2014 [7]. Wald and Arrol investigated in an uncontrolled before and after study the impact of the introduction of animation-supported consent in a UK Cardiac Centre where only paper-based materials had been used previously. They compared the two years immediately preceding the implementation on the animations on complaints or serious incidents that were reported and found to be due to failure to inform participants. The introduction of the animation-supported consent was associated with a 70% reduction in complaints or serious incidents [8]. Whilst that study was related to consent to a standard clinical procedure, the issue of retention of those in trials is always a concern. A further narrative review in 2021 of video interventions used for patient information and education found that targeted video-based interventions can improve patient experiences and outcomes [9].

Moe-Byrne et al. have suggested after the evidence was evaluated in 2022 that the use of animations is a more effective means to deliver education/information compared with traditional paper-based information sheets, although the authors commented that the evidence base was highly variable and mostly based on small trials [10]. However, there are some large standalone studies—one such study comes from the USA, where 1194 cancer patients and survivors took part in a four-arm experiment to assess the effects of EAs compared with text-only brochures, brochures with visuals and the materials that were currently in use by the National Institute for Health (NIH) [11]. That study showed that EAs improved participants’ knowledge about and attitudes toward clinical trials and were more effective than any of the brochures, especially for those individuals with low motivation and low literacy to comprehend health-related information. Small standalone and embedded trials and a meta-analysis have also shown that the use of multimedia information can make information about trials easier to understand and may increase trial recruitment rates [12–15]. However, a recent meta-analysis of 5 studies within a trial (SWATs) showed that multimedia alongside written information did not improve trial recruitment rates [16].

Alongside the development of tools such as EAs to facilitate information dissemination to potential participants, it should be recognised that the quality and quantity of information provided to help individuals make informed choices about trials has also been evolving. There have been national initiatives in the UK regarding improving the promotion of trials and their information. One of the largest funders, the National Institute for Health and Care Research (NIHR) has created online platforms to highlight trials and research opportunities https://bepartofresearch.nihr.ac.uk/. There has also been the launch of Patient Information Sheet standards [17] and the publication of NIHR INCLUDE Guidance [18] which highlights the importance of offering trials as widely as possible through acknowledging, appreciating and being able to address differences.

The aim of this study was to collect information on current practice in UKCRC Clinical Trials Units on the use of explainer animations and to determine any barriers to their use.

## Methods

The survey results reported here are part of a bigger project—the EXPLAIN initiative (https://explain.octru.ox.ac.uk/). The initiative has three parts:

- To understand the current usage of explainer animations (EAs) across the UKCRC—a national network of academic clinical trials units (CTUs) in the UK that have been assessed by an international panel of experts in clinical trials research, and who undertake the majority of academic-led clinical trials in the UK. https://ukcrc-ctu.org.uk/
- To survey the wider clinical trial community to seek their input into which topics should be created as explainer animations for wider use.
- To create some fully accessible explainer animations that will be available to use across clinical trials and initiate a repository for these animations.

The project team included trialists, methodologists and clinicians from three CTUs in England and Wales registered with the UK CRC CTU Network. The group designed a cross-sectional survey to elicit information on the use of explainer animations within UKCRC CTUs (Supplementary Material 1).

The survey was accessible online in Microsoft Forms and a link to the survey was emailed via the UKCRC CTU network on 1st February 2023 to all 52 registered CTUs Directors and Trial Management Leads. Generic reminders were sent out on the 22nd of February and 3rd of March 2023 to the same recipients to encourage a response, and the survey deadline was extended until the 7th of March 2023. Additionally, where a response had not been received from a unit, targeted emails were also sent to individuals within the unit if any of the authors had contacts there.

The survey did not require a university ethical review as it was considered a service evaluation.

## Results

Forty-one responses from 35 registered CTUs were received, representing a CTU response rate of 67%. Responses were received from across all 4 nations of the UK, including responses from both generalised and specialist area CTUs including in the areas of perinatal health, diabetes, cancer, blood and transplantation. Four CTUs provided multiple responses (three different responses from two CTUs; two responses from another two CTUs), and we pragmatically took the first response received from the CTU. It should be noted that where there were multiple responses—both respondents in all cases provided the same information on whether their CTU had used explainer animations or not.

Of the 35 CTUs, 24 (69%) had used at least one explainer animation in their trial portfolio in the last five years. Of these, only two CTUs were using explainer animations on more than half of their portfolio of trials. The majority (75%) were only using explainer animations on a small proportion of their portfolios: 54% on less than 10% of trials in their portfolios and 21% on 10–25% of their portfolio.

Of the 11 CTUs that had not used any explainer animations in the past five years, the main reason cited was lack of expertise (Table 1). Two CTUs also reported that they had no need for explainer animations for the types of trials that their CTU delivers; one of these CTUs expanded upon this by stating:

They aren’t applicable for all trial designs but would definitely be of benefit when explaining difficult concepts or to have for general information on trial website etc. (CTU Response 11)

**Table 1:** Reasons for not having used any explainer animations

*Reasons for not having used any explainer animations to date (multiple reasons could be selected)*	*Number of CTUs selecting this option (n* = *11)*
*Lack of expertise*	8
*Costs/no funds available to do so*	6
*Lack of resources (i.e. time)*	5
*Had not considered it before*	4
*Had no need to use them for the type of trials the CTU delivers*	2

The three main reasons given for using explainer animations (Table 2) were to explain a trial-specific aspect, e.g. the treatment/intervention (92% of CTUs), the premise (background) of a trial (88% CTUs) and explain generic trial concepts such as randomisation or blinding (58% CTUs).

**Table 2:** Reasons for using an explainer animation

*Reasons for using an explainer animation (multiple reasons could be selected)*	*Number of CTUs selecting this option (n* = *35)*
**Trial-specific**	**44**
* A trial-specific aspect, e.g. the treatment/intervention*	22
* The premise (background/scientific rationale) of a trial*	21
* Patient and carer education for home care*	1
***Generic***	**15**
* Generic concepts, e.g. randomisation, blinding*	14
* Electronic data capture system*	1

Some of the respondents provided free text information to give context to their responses, five of these are included below:

Many varied topics have been the subject of animations including in obstetric trials, sepsis, challenging behaviour and learning difficulties, and the use of routinely collected data. There was also a variety of reasons for their development, including supported the patient information sheet (PIS), recruitment, explaining complex topics and explaining randomisation. (CTU Response 9)

One video was developed for a paediatric trial aimed at the child but to be watched with a parent/guardian and covered usual care and intervention and what would happen to children taking part in the study. For the same study, other videos were developed to show the use of equipment at home. (CTU Response 12)

Explainer animations have been developed with three different audiences: 1. Potential trial participants to explain the concept of the specific trial concerned, 2. Trial participants about the management of a treatment side-effect, and 3. Participating sites explaining the context of the trial and why this is an important research question to address. (CTU Response 30)

Some were developed to supplement the Patient Information Sheet. Others were developed to explain concepts such as pharmacokinetic studies and why they’re needed, or the MAMS [Multi-Arm, Multi-Stage] design. (CTU Response 31)

We have developed several trial-specific animations that briefly explain the disease area, why the trial is being done, what the intervention is, and hopes for the outcome of the trial. We have also produced a few videos featuring interviews (e.g. with the Chief Investigator) talking about a trial. (CTU Response 29)

The specifics of where EAs were utilised in trials and the average length of those utilised are listed in Table 3. EAs were used most often prior to any consent discussions and the most used were between 2 and 3 min in length, with a range from 1 to 10 min. Only one unit was using an explainer shorter than 2 min, and one unit an explainer lasting between 5 and 10 min.

**Table 3:** Details of those EAs utilised by CTUs

*Timepoint of using explainer animations to date* *(multiple reasons could be selected)*	*Number of CTUs selecting this option (n* = *24)*
*Prior to the consent discussion*	18
*During the consent discussion*	12
*After the consent discussion*	14

Two thirds (66%) of those using EAs stated that they always had voiceovers on their videos, whilst the remaining third had a mixture of voiceovers or no voiceovers on their animations. Fifteen of the 24 CTUs reported that the animations they had created to date had solely been in English (Table 4).

**Table 4:** Languages used to date in explainer animations

	***Number of CTUs selecting this option (n*** ** = ** ***24)***
*Only English* ^*a*^	15
*English and another language(s)*	9
*Hindi and/or Hindi subtitles*	3
*Polish and/or Polish subtitles*	2
*Bengali subtitles*	1
*Filipino subtitles*	1
*Romanian subtitles*	1
*Turkish subtitles*	1
*Panjabi*	2
*Malayalam*	1
*Norwegian*	1
*Portuguese*	1
*Spanish*	1
*Urdu*	1
*Other language not explicitly stated*	1
*Welsh* ^*b*^	1

^a^11 of the units had incorporated English subtitles

^b^3 Welsh CTUs responded to this survey, of which 2 had to date used an EA

Three had used subtitles on all their animations, 12 stated they used subtitles on some of their animations and nine stated they did not use subtitles at all on their animations.

Of the 24 CTUs using EAs, only four were currently reviewing usage statistics for all the videos they had produced. A further ten did monitor the use of some of their EAs created but not all, and the remaining ten did not monitor any usage at all. Of the 14 CTUs using some form of monitoring (the types of monitoring can vary from page views, statistics from YouTube/Vimeo, starts/stops/duration—if implemented by the CTU programming team):

- One research team recorded if they had used the EA.
- Four reviewed metrics (e.g. page views) from their trial/CTU web pages.
- Seven used either one of or a combination of YouTube or Vimeo/YouTube views or Google Analytics.
- Two checked viewing figures—but did not cite through which tools, of which one CTU broke this down into the number of unique viewers and the number of views in each language and the percentage of individuals watching until completion of the videos.

One of the teams further commented that when videos were shared with recruiting sites as stand-alone videos—they had not to date always recorded the usage of them. Only one team had also explicitly stated that they had undertaken specific surveying of participants about their opinions on the videos as a separate spin-off study.

The usage of the statistics was questioned. Eleven of the 14 units collecting these responded on how the figures were used. The reasons included to determine whether the video was helpful in getting people interested in a trial, to get a sense of how many times a video is watched and for how long, for Trial Steering Committee/Trial Management Group/Funder updates, and to be added to communications and engagement trackers. Three CTUs reported that they had utilised metrics so that groups could gauge the reach of the videos, for new trials (which are run remotely) to look at the relationship between mailed invitations and visits to the trial website/recruitments. One CTU reported that they surveyed the research team about using the videos.

Respondents who were willing to give an approximate cost for each animation produced showed the most common cost incurred was less than £5000 (Table 5). Costs were routinely included in grant applications in about two-thirds of CTUs (15 Units), 10 units stated that they routinely include costs for EAs, 5 units said they sometimes do, and 3 stated they did not.

**Table 5:** Average costs of explainer animations excluding UK Value Added Tax (VAT)

	***Number of CTUs selecting this option (n*** ** = ** ***24)***
*No cost*	5
*Less than £5000*	9
*£5000–£6000*	5
*£6000–£7000*	0
*Greater than £7000*	1
*Prefer not to say*	4

Note VAT is Value Added Tax and in the UK this is currently an additional 20% added onto the cost a product/service

Sixteen CTUs (66%) had developed explainer animations in-house. Cost was the biggest driver for utilising in-house development either due to prohibitive prices from external providers or a lack of funding in the grant budget. Other reasons cited were speed of delivery, in-house expertise availability and value for money.

In terms of branding, 14 units (58%) did not use any branding in the EAs produced, whilst nine used trial logos and one CTU used their unit branding only. There did not seem to be any unit level guidance on uniformity and acknowledgements of those who had produced the videos.

Free text comments from the CTUs received are included below. They all were positive in regard to the provision/development of videos/EAs:

I’m interested to see more examples of videos that attempt to engage patients and the public with trial concepts generally - participation in trials, how they work, ethos and rationale, what randomisation is, what masking is, what controls are, etc etc, animations, dramatisations - I really love the NIHR “COVID and me - be part of research” series of videos. (CTU Response 28)

The use of short explainer animations would be really welcomed in terms of improving access to clinical trials. It would help if these have a consistent look and feel, but effective patient & public advocate input is more important and using a diverse range of voices to ensure that fears and concerns about research and trials can be considered….. our explainer animations to date have been developed for specific trials. Access to very short (e.g. >1min) videos about key concepts (e.g. consent, randomisation) would be very useful. (CTU Response 30)

Generic videos to explain broad trial principles would be useful for participants, site staff and CTU staff. (CTU Response 37)

There are many general aspects of clinical trials (e.g. randomisation) where having access to a library of videos could be useful for trial teams and recruiting sites. We do not have the resource to produce our own library of this kind. (CTU Response 29)

We have discussed the use of video or cartoon clips with out PPI groups and they are well received. They aren’t applicable for all trial designs but would definitely be of benefit when explaining difficult concepts or to have for general information on trial web sites etc. (CTU Response 11)

## Discussion

Participant information and understanding is a key expectation of Good Clinical Practice [19]. Poor understanding can result in lower recruitment and reduced retention (when individuals may withdraw from or become lost to follow-up if they did not fully understand what would be expected from them). Potential participants need to have all the facts about a trial and what it entails delivered in a manner that is easily understood and inclusive to all. Accessibility in animations is key. In this survey, 63% were only producing EAs in English, within the UK there is a diverse range of languages spoken—those listed in the 2021 UK Census as the top other languages spoken other than English and Welsh were Polish (1.1%), Romanian (0.8%) and Panjabi (0.5%) this did not align with the EAs reported and we should be cognizant of the population trials are looking to recruit from and the changing diversity in the UK [20]. Subtitling had been included in nearly two-thirds of animations (63%); as this data was collected before the release of “Participant Information Quality Standards” in the UK [17], which advises that all video information should be subtitled, this is an encouraging baseline for the trials community to grow from.

Explainer animations have the potential to aid understanding of clinical trials [10]. It is reassuring to see over half of the registered academic clinical trials units in the UK are already embracing their potential—either in the core generic concepts that are inherent in most clinical trials (e.g. randomisation, what clinical trials are, who performs clinical trials, what oversight trials have) or in the specifics of individual trials (e.g. the background to a funded trial or the specifics of the interventions involved). This is promising, as a survey of UKCRC CTUs back in early 2018 found only 18 out of 24 responding CTUs had some digital tool experience in recruitment [6]. It is encouraging that in this survey this number is now 24, although this figure should be viewed with caution as neither this nor the referenced survey received a 100% response. However, due to anonymity of the 2018 survey results we are unable to see if the additional numbers are different CTUs or perhaps an additional 6 units. We do need to be mindful of sharing good practice within the research community and not ‘reinventing the wheel’ regarding animations that could be shared widely across CTUs—this was echoed in the free text comments from the units. There is a definite unmet need for generic freely available animations which promote understanding of key concepts so that trial resources can be allocated to more specific animations for individual trials.

Barriers to the implementation of EAs by CTUs were reported as alack of resources and costs to produce them, it is hoped that the data shown here will address the cost issue and will encourage more CTUs to include the costs for generating EAs in future grant requests. Doing so will aid in addressing the suggestions of the NIHR INCLUDE project [18] and with the use of subtitles will increase inclusivity across minorities within the population. As part of the EXPLAIN project, we will also be producing guidance on the process of creating Explainer animations with the aim of demystifying the process.

Some hospital trusts have reported on matrices of quick response (QR) codes added to information sheets about interventions commonly delivered—that link either to documentation about a procedure [21] or educational videos about procedures [22]. It would be efficient to instigate a similar QR matrix to signpost potential participants to common concepts in animations that have been co-developed by both trial groups and the public.

The strengths of this study are that the responses include CTUs from England, Scotland and Wales, providing the first insight into the usage of EAs in UK-wide clinical trials and the current costs of creating EAs. The principal weakness is in the number of respondents to the survey. We also did not survey units on the size of their unit and the number of individuals that work within it—which may have affected the ability to have the capacity and experience of staff to deliver the creation of EAs. However, we do know which units replied to the survey, comparing the online portfolios of the units, there was an approximate 50:50 response from both large well-established CTUs and newer smaller CTUs.

## Conclusions

This survey reports on the usage of explainer animations in UKCRC CTUs since 2018. It is encouraging that the survey showed that 69% of the CTUs that replied were starting to use this technology, the 24 that stated they were equated to approximately half of the registered CTUs. Most animations to date seem to be very trial-specific; the EXPLAIN initiative aims to produce a set of freely available explainer animations on generic concepts relevant to most trials, e.g. randomisation and informed consent. On the launch of these animations, there will be a call to CTUs if any are willing to share any of their generic animations which will then be added and available at https://explain.octru.ox.ac.uk.

### Supplementary Information

**Supplementary Material 1.**

## Data Availability

The survey used is available as an appendix to the paper. The datasets used and/or analysed during the current study are available from the corresponding author on reasonable request.

## Abbreviations

CTU: Clinical trials unit
EA: Explainer Animation
NIHR: National Institute for Health and Care Research
UKCRC CTU: United Kingdon Clinical Research Collaboration – Clinical Trial Units
VAT: Value Added Tax
