# Supplementary material for: Current usage of explainer animations in trials: a survey of the UKCRC registered clinical trial units in the UK
Source: Trials. 2024 Mar 28;25:224. doi: 10.1186/s13063-024-08060-6 (PMC10976673; doi:10.1186/s13063-024-08060-6)
Supplement: Supplementary file 1 — Supplementary Material 1. [file 13063_2024_8060_MOESM1_ESM.pdf]

EXPLAIN initiative: CTU survey on explainer videos - version 1.0_10Jan2023

* Required

Welcome to the EXPLAIN CTU Survey

The EXPLAIN study will seek the views of those who take part in clinical trials and those involved in designing, managing, and recruiting to clinical trials to help identify the key topic areas for which explainer videos should be created. This information will then be used to start the creation of a library of explainer videos that will be freely shared with UKCRC registered CTUs to utilise in and across their portfolio of trials as each trial/ CTU decides to.

As part of the EXPLAIN project, we would also like to understand on a CTU level, what experience you have of using explainer videos/animations. You can read more about the project at https://explain.octru.ox.ac.uk

The survey should take no more than 10 minutes to complete.

Thank you in advance for your time and participation.

EXPLAIN CTU survey Privacy Notice

How will my data be used?
The data we will collect that could identify you will be your e-mail address and CTU.
We will take all reasonable measures to ensure that data remain confidential.
The responses you provide will be stored on secure servers (accessible only by members of the research team) and may be used in publications and conference presentations. Identifiable information will be deleted as soon as it is no longer required for the research. Research data will be stored for a minimum of 3 years after publication or public release of the work of the research.

Who will have access to my data?
The University of Oxford is the data controller with respect to your personal data and, as such, will determine how your personal data is used in the research. The University will process your personal data for the purpose of the research outlined above. Research is a task that we perform in the public interest. Further information about your rights with respect to your personal data is available from https://compliance.admin.ox.ac.uk/individual-rights.

The data you provide may be shared with members of the research team at Cardiff University and the University of Nottingham.

1. Before starting the survey, please confirm that you have read and agree to the data Privacy Notice statement above *

◯ Yes

2. Name of CTU *

- ○ Barts CTU
- ○ Barts & The London Pragmatic CTU
- ○ Birmingham CTU
- ○ Bristol Trials Centre
- ○ CaCTUS
- ○ Cambridge CTU
- ○ Centre for Trials Research, Cardiff
- ○ CHaRT
- ○ Comprehensive Clinical Trials Unit at UCL
- ○ CRCTU (Birmingham)
- ○ CRUK & UCL Cancer Trials Centre
- ○ Derby CTSU
- ○ Diabetes Trials Unit Oxford
- ○ Glasgow CTU
- ○ ICNARC
- ○ ICR-CTSU
- ○ Imperial CTU
- ○ Keele CTU
- ○ Leeds CTRU
- ○ Leicester CTU
- ○ Liverpool Trials Collaborative
- ○ King's Clinical Trials Unit at King's Health Partners
- ○ London School of Hygiene & Tropical Medicine CTU
- ○ Manchester CTU
- ○ MRC CTU
- ○ Newcastle CTU
- ○ NHS Blood and Transplant CTU
- ○ Northern Ireland CTU
- ○ Norwich CTU
- ○ Nottingham CTU
- ○ NPEU CTU
- ○ NWORTH
- ○ Oxford Clinical Trials Research Unit (OCTRU)
- ○ Oxford CTSU
- ○ Oxford Primary care and Vaccines Collaborative CTU
- ○ Papworth Trials Unit Collaboration
- ○ Peninsula CTU
- ○ PRIMENT CTU at UCL
- ○ Royal Marsden CTU
- ○ Sheffield Clinical Trials Research Unit
- ○ Southampton CTU
- ○ Surrey CTU
- ○ Swansea Trials Unit
- ○ Tayside CTU
- ○ Warwick CTU
- ○ York Trials Unit
- ○ Other

3. E-mail address of person completing survey *

CTU use of explainer videos

For the purposes of this questionnaire - an explainer video is a very short video/animation which helps people to understand more about taking part in a trial, whether it is about the premise of a trial or about a particular part of a trial or about something general related to clinical trials such as 'What is randomisation?'.

4. In the last 5 years, have you used any explainer videos in any trials in your portfolio? *

- ◯ Yes
- ◯ No

5. In the last 5 years, what proportion of the trials in your portfolio used explainer videos? *

- ◯ <10%
- ◯ 10-25%
- ◯ 26-50%
- ◯ >50%

Exploring why you have not used explainer videos

6. Please indicate the reason(s) you have not used explainer videos *

- [ ] Cost/No funds available to do so
- [ ] Lack of expertise
- [ ] Lack of resource
- [ ] Had no need to use them for the type of trials the CTU delivers
- [ ] Had not considered it before
- [ ] Other

7. Were any of the following reasons for not using explainer videos to date? *

	Yes	No
We did not have a need to for the types of trials delivered	◯	◯
Would have liked to, but did not have resource to produce	◯	◯
Have not considered using them	◯	◯

About the explainer videos developed by your CTU

8. Please specify whether the explainer videos covered any of the following (tick all that apply) *

- [ ] Generic concepts e.g. randomisation, blinding
- [ ] The premise of a trial
- [ ] A trial-specific aspect e.g. the treatment/intervention
- [ ] Other

9. Please provide further details about what explainer videos were developed for/what topic(s) they covered

10. What languages have you produced explainer videos in?

11. Do you "brand" your explainer videos so they have a consistent look and feel across the study or CTU? *

- [ ] Yes, branded across the study
- [ ] Yes, branded across the CTU
- [ ] No
- [ ] Other

12. Please provide any further information about video branding (if relevant) below

13. Do your explainer videos have a voiceover? *

- ○ Yes - all
- ○ Yes - but only some do
- ○ No

14. Do you record any usage statistics? *

- ○ Yes - for all
- ○ Yes - but only for some
- ○ No

15. How are these recorded?

16. How do you use these statistics?

17. On average, how long are the explainer videos you have used? *

- ◯ <1 minute
- ◯ 1-2 minutes
- ◯ 2-3 minutes
- ◯ 3-4 minutes
- ◯ 4-5 minutes
- ◯ 5-10 minutes
- ◯ 10+ minutes

Costs of your explainer videos

18. On average, what is the cost for each explainer video you have produced (excluding VAT)? *

- ◯ No cost
- ◯ Less than £5,000
- ◯ £5,000 - £6,000
- ◯ £6,000 - £7,000
- ◯ More than £7,000
- ◯ Prefer not to say

19. Do you include the cost of explainer videos in your grant applications? *

- ◯ Yes - always
- ◯ Yes - only sometimes
- ◯ No
- ◯ Other

20. Please detail source(s) of funding used to develop explainer videos *

The development of your explainer videos

21. Have any of your explainer videos been developed in-house? *

- ◯ Yes
- ◯ No

22. Please detail reason(s) for producing in-house rather than using an external provider

23. Have any of your explainer videos been developed by an external provider? *

- ◯ Yes
- ◯ No

24. Please detail reason(s) for using an external provider rather than developing in-house?

Sharing explainer videos

25. Would you consider sharing your explainer videos with the EXPLAIN team? *

- ◯ Yes
- ◯ No
- ◯ Maybe

26. Please provide details of person to contact about this

[ ]

Use by participants

27. Where do participants access your explainer videos from? *

- [ ] On trial website
- [ ] On CTU website
- [ ] YouTube
- [ ] Vimeo
- [ ] Facebook
- [ ] Twitter
- [ ] Other

28. Typically, when are the explainer videos first introduced to participants (select all that apply) *

- [ ] Prior to the consent discussion
- [ ] During the consent discussion
- [ ] After the consent discussion

29. Do any of your explainer videos use subtitles? *

- ○ Yes - all of them
- ○ Yes - but only some do
- ○ No

30. In what languages have subtitles been used?

Additional Comments

31. Would you like to have access to a library of explainer videos to use in your trials? *

- ◯ Yes
- ◯ No
- ◯ Maybe

32. Why do you think this wouldn't be of use to your CTU?

33. Please add any comments that you wish to share from your CTU about explainer videos below

This content is neither created nor endorsed by Microsoft. The data you submit will be sent to the form owner.

Microsoft Forms
